# Processing and Characterization of Poly(lactic acid) (PLA) Films Containing Pomegranate Peel Powder

**DOI:** 10.3390/polym18020274

**Published:** 2026-01-20

**Authors:** Ömer Faruk Uslu, David Krieg, Benedikt Theodor Hiller, Özge Taştan Ülkü, Nebahat Aral

**Affiliations:** 1 Institute for the Circular Economy of Bio:Polymers, Hof University (IBP), 95028 Hof, Germany; 2 Department of Materials Science and Nanotechnology Engineering, Faculty of Engineering and Natural Sciences, Yeditepe University, Ataşehir, 34755 İstanbul, Türkiye; 3 Department of Food Engineering, Faculty of Engineering and Natural Sciences, Yeditepe University, Ataşehir, 34755 İstanbul, Türkiye; ozge.tastan@yeditepe.edu.tr

**Keywords:** Poly(lactic acid) (PLA), pomegranate peel powder (PoP), biodegradable composites, bioactive additives, melt extrusion, film characterization

## Abstract

The present study analyses the changes in antioxidative behavior of biodegradable Poly(lactic acid) (PLA)-based composite films with bioactive additives derived from pomegranate peel, an abundant agricultural by-product rich in antioxidants and antimicrobials. PLA-based composites were prepared by incorporating industrial-grade pomegranate peel powder (PoP) via melt extrusion at concentrations of 1–5 percent by weight (wt.%). For mechanical characterization, the resulting films were subjected to tensile testing. Their thermal properties were further characterized using thermogravimetric analysis (TGA), differential scanning calorimetry (DSC), dynamic oxidation induction temperature measurements (OIT), complemented by Fourier-transform infrared spectroscopy (FT-IR), color analysis, rheology, scanning electron microscopy (SEM), and UV-Vis spectroscopy. Results show that the incorporation of PoP had no significant impact on the characteristic transition temperatures (Tg, Tm, and Tc) of PLA, indicating that the thermal behavior of the polymer matrix was largely preserved. However, while the thermo-oxidative stability of PLA was improved in the presence of PoP, with a maximum at 3 wt.% of PoP, increasing the OIT by 30 °C, the mechanical performance of the composite films was adversely affected, as evidenced by decreased tensile strength and elongation at break indication embrittlement, especially for ≥3 wt.% of PoP. Significant changes were observed in the films’ surface properties, as well as in their color parameters and UV transmittance values. Consequently, while PoP offers potential bioactive functionality for use as a sustainable additive, its content must be carefully optimized to maintain an acceptable balance between functionality and mechanical integrity.

## 1. Introduction

Plastic pollution is one of the most pressing environmental challenges of our time. Mainly derived from finite petrochemical resources, globally, 400 million tons of plastic are produced annually, of which only a small fraction of 14% is recycled effectively [1]. This results in massive waste accumulation, with an estimated 22 million tons of plastic entering natural ecosystems in 2019 alone [2], not only increasing the consumption of non-renewable resources but also endangering the environment through the release of toxic additives [3].

Addressing these challenges, biodegradable polymers from renewable resources have emerged as promising alternatives. Among these sustainable materials, polylactic acid (PLA) stands out as a leading candidate, as it offers additional advantages such as reduced energy consumption by 25 to 55% during production compared to fossil-based plastics [4]. PLA shows great potential for use in various industries, with food and beverage packaging currently representing the main industrial application for PLA [5]. As the global demand for sustainable materials grows, biodegradable polymers like PLA offer a viable solution to plastic pollution and support the transition towards a circular economy by integrating renewable resources and reducing environmental impacts. However, PLA still faces challenges such as brittleness and limited stability, which can be overcome through advances in material blending, processing techniques, and the use of additives [5,6,7].

Biogenic materials are particularly attractive for the latter approach. Ashori et al. [8] reinforced polypropylene with wheat straw and rice husk as sustainable alternatives to wood fibers, improving the mechanical properties, especially with a coupling agent [8]. Valdés et al. [9] investigated biocomposites based on poly(ε-caprolactone) (PCL) and almond skin, an agricultural by-product. The addition of almond skin improved the modulus of elasticity, increased crystallinity, and reduced enzymatic degradation rates [9]. Xie et al. [10] developed biodegradable and active food packaging films from potato peel with bacterial cellulose and curcumin. The films showed enhanced tensile strength, reduced water vapor permeability, and effective antioxidant capacity. As a result, lipid oxidation of stored fresh pork was significantly reduced, demonstrating a sustainable approach for food preservation [10].

Pomegranate peel accounts for about 30–40% of the fruit, is often discarded as a by-product of juice production, and is rich in polyphenols, which have remarkable antimicrobial and antifungal activities [11,12,13]. Blueberry extracts not only prevent oxidative degradation of packaged products but also inhibit foodborne pathogens [11,14]. Grapefruit, with its oils rich in limonene and bioactive compounds like flavonoids, offers potent antimicrobial properties while utilizing waste peels effectively [11,15]. Consequently, packaging materials, such as plastic films, can be given antimicrobial and antioxidant functionalities by incorporating oils, extracts, and peels from fruits such as pomegranate, blueberry, and grapefruit, not only avoiding waste from landfills but also contributing to extended product shelf life and improved food safety [11,16,17].

Previous works on PLA filled with pomegranate focus on antimicrobial activity using laboriously produced pomegranate peel extract in solvent-casted PLA films or electrospun PLA/HPMC mats [18,19], and on PLA/starch composites, where peel powder primarily serves as an antimicrobial/reinforcing phase [20]. In contrast, the present work targets the more practical approach of incorporating industrial-grade pomegranate peel powder (PoP) into neat PLA films using solvent-free melt extrusion and analyzing its potential as a natural antioxidant with respect to commercial film production. Therefore, the PLA-based materials are (i) produced at realistic processing temperatures (~195 °C) that can affect the bio additive chemistry, (ii) analyzed for improvements in thermo-oxidative stability, and (iii) their mechanical properties are linked to processing- induced molar-mass changes and filler dispersion. In addition, optical coloration and UV-blocking are investigated as application-relevant characteristics to establish PoP as a possible natural antioxidant and colorant for PLA film by closing the research gap in processing–structure–property relationships and determining optimal filler contents for balancing stabilization, appearance, and mechanics of PLA-based biocomposite films.

## 2. Materials and Methods

### 2.1. Materials

Characterized by a density of 1.24 g/cm^3^, a melting peak temperature (Tm) of 155 °C, a glass transition temperature (Tg) of 60 °C, a tensile strength of 45 MPa, and an MFI of 3 g/10 min at 190 °C and 2.16 kg, PLA Luminy^®^ LX175 was purchased from Total Corbion PLA BV (Gorinchem, The Netherlands). This PLA grade is suitable for use in food contact applications. Industrial pomegranate peel (PoP) waste was supplied in powder form from DOHLER (Izmir, Türkiye), derived from commercial pomegranate juice production. It was obtained by drying the waste remaining at the end of pomegranate processing with a hot air dryer (60–65 °C for 4–5 h) and then grinding it to an average size of approx. 6.5 µm (D50). No bleaching or chemical pretreatment was applied.

Proximate composition of PoP was ~6–7 wt.% of moisture (before drying), ~3–4 wt.% of protein, ~4–5 wt.% of ash, ~17 wt.% of crude fiber, ~0.8 wt.% of fat, and ~66–67 wt.% of carbohydrates [21,22]. PoP is rich in antioxidants, polyphenols, tannins, pectic polysaccharides, and lignocellulosic matter [23,24,25].

### 2.2. Processing of PLA Films

The PLA pellets were predried at 60 °C for at least 8 h in a BIN S 15 with a LUXOR 50 dry air generator (Motan Holding GmbH, Konstanz, Germany). The PoP was dried in a Memmert UF450plus (Memmert GmbH Co. KG, Schwabach, Germany) at 60 °C overnight to achieve a moisture content of ~3% to minimize hydrolytic degradation. The moisture content of PoP was determined via a Kern DBS60-3 (KERN Sohn GmbH, Balingen, Germany). The dry PLA pellets were dry-blended with the dried PoP in ratios of 1, 2, 3, and 5 percent by weight (wt.%). Dry-blending was performed by manual tumble mixing in closed containers (2–3 min tumbling) and directly fed into the extruder.

The mixed materials were extruded using a Composer 450 miniaturized single-screw extruder equipped with a mixing screw (3D Evo B.V., Utrecht, The Netherlands). The screw diameter of the extruder was 20 mm, and it had a L/D-ratio of 15:1. The miniaturized single-screw extruder was operated with modifications, and the details were thoroughly documented in an earlier study [26]. The four heating zones of the extruder were set to 190, 200, 195, and 180 °C from the first (hopper) to fourth (die) zone. The turning speed of the screw was 6 rpm. The minimum residence time was around 12 min, and the maximum residence time was up to 34 min. Mass throughput was around 240 g/h. No degassing during processing was used. The diameter of the die was 2 mm. The resulting biocomposite filaments were obtained at the end of the extrusion process and cut into pellets.

The PLA-based biocomposite pellets were hot-pressed with a hydraulic laboratory press P 200 S (VOGT Labormaschinen GmbH, Berlin, Germany) to form films. Sample masses of 4 g of PLA pellets were placed between two polytetrafluoroethylene sheets for easier removal. The pressing was conducted in three consecutive phases. The first phase took 90 s with a pressure of 2 bar, a temperature of 195 °C, and an opening gap of 2.5 mm. The time of the second phase was 30 s, at a pressure of 120 bar, a temperature of 195 °C, and an opening gap of 0.3 mm. In the third phase, a pressure of 200 bar and a temperature of 195 °C were applied for 90 s. After pressing, the films were rapidly cooled between two metal plates. With this last step, films containing 0, 1, 2, 3, and 5 wt.% PoP and with an average thickness of 0.11, 0.09, 0.08, 0.10, and 0.10 mm, respectively, were obtained. The resulting films were named PLA ref, PLA 1% PoP, PLA 2% PoP, PLA 3% PoP, and PLA 5% PoP with respect to the PoP content wt.%.

The PLA-based biocomposite pellets were used for differential scanning calorimetry (DSC), thermogravimetric analysis (TGA), dynamic oxidation induction temperature (OIT), and rheology analysis, while color measurements, Fourier-transform infrared spectroscopy (FTIR), and tensile testing were performed with the produced films.

### 2.3. Film Characterization

Fourier-transform infrared spectroscopy analysis (FTIR) of PLA films was conducted with a Nicolet IS50 ATR from Thermo Fisher Scientific Inc. (Waltham, MA, USA). All the PLA films were analyzed in a frequency range from 400 to 4000 cm^−1^. Measurements were taken from three different areas of each sample.

Differential scanning calorimetry (DSC) measurements were performed on a Netzsch DSC Polyma 214 (NETZSCH-Gerätebau GmbH, Selb, Germany). Each measurement consisted of two cycles with a heating run from 0 to 220 °C and a cooling run from 220 to 0 °C at 10 K/min in a nitrogen atmosphere with a sample mass of 5.0 ± 0.5 mg. The DSC derived glass transition temperatures (Tg) carry an accuracy of ±2 °C, while the melting (Tm) and cold-crystallization (Tcc) temperatures have an accuracy of ±1 °C.

Dynamic oxidation induction temperature (OIT) measurements were also conducted using a Netzsch DSC Polyma 214 (NETZSCH-Gerätebau GmbH, Selb, Germany). A sample mass of 5.0 ± 0.5 mg was placed in an open aluminum crucible. The temperature was increased from 25 to 450 °C at a constant heating rate of 20 K/min in a 100% oxygen atmosphere at a flow of 50 mL/min. The dynamic OIT was determined by the offset method with a delta of −0.15 W/g to the baseline. The temperature measurement accuracy for OIT determinations was approximately ±2 °C.

For thermogravimetric analysis (TGA), a Netzsch TG 209 F3 Tarsus (NETZSCH-Gerätebau GmbH, Selb, Germany) was utilized. The measurements were performed in a temperature range from 60 to 700 °C with a heating rate of 10 K/min and sample masses of 5.0 ± 0.5 mg in both nitrogen and oxygen environments. For determining the mass loss, the 100% sample mass was obtained at 120 °C to consider water evaporation. Each sample was measured in two sets to ensure the reliability of temperature values.

For tensile testing, the ASTM D882 standard was followed [27]. Rectangular tensile specimens with a gauge length of 50 mm, width of 15 mm, and thickness of 0.08–0.11 mm were tested at a pulling speed of 10 mm/min. Tests were performed using the Instron 5966 (Norwood, MA, USA). A minimum of six measurements was conducted for each sample. The stress and strain values were expressed as average values, considering the standard deviations of the data.

The color characteristics of the samples were quantitatively evaluated using a Minolta colorimeter (Model CM-5, Konica Minolta, Tokyo, Japan), and the results were expressed as L* (lightness), a* (redness/greenness), and b* (yellowness/blueness). Values were gathered in triplicate and averaged. Chroma (C*) was calculated using Equation (1), and Hue angle (°Hue) was calculated using Equation (2). The total color difference (ΔE) was calculated compared to the PLA reference sample, and values greater than 5 were considered to represent perceptible color differences visible to the human eye [28]:

(1) $$C^{*}={\left(a^{2}+b^{2}\right)}^{\frac{1}{2}},$$

(2) $${}^{\circ}Hue={tan}^{-1}\left(\frac{b}{a}\right)$$

Oscillation rheology measurements were performed using an HR 20 rheometer from TA Instruments (New Castle, DE, USA) with a 25 mm plate–plate geometry. Two frequency sweeps at 130 and 180 °C were performed from 0.1 to 629 rad/s in air. From the two sweeps, the master curve was created using the TRIOS software (version 5.5.0.232), also provided by TA Instruments (New Castle, DE, USA). Prior to the frequency sweeps, a strain sweep was conducted to ensure measurements were performed on the Newtonian plateau.

For the scanning electron microscope (SEM) analysis, films were cut into small pieces and then coated with a thin gold layer for 30 s to minimize surface charging. Imaging was performed on a Thermo Scientific Phenom XL SEM (Waltham, MA, USA) operated at an accelerating voltage of 5 kV. Micrographs were acquired at nominal magnifications of 500× and 2500× at room temperature.

An Agilent 8453 UV–Vis spectrophotometer (Santa Clara, CA, USA) was used to measure the UV-Vis spectroscopy of PLA films in the 280–1000 nm range.

The analysis results were assessed using the SPSS software (version 27) program (IBM SPSS Statistics, Armonk, NY, USA) for statistical analysis. The normality of data distribution was assessed using the Shapiro–Wilk test before statistical analysis. A significance level of *p* < 0.05 was applied in the evaluation, using one-way ANOVA and Duncan’s multiple comparison methods.

## 3. Results and Discussion

### 3.1. FTIR Results

**Figure 1:**
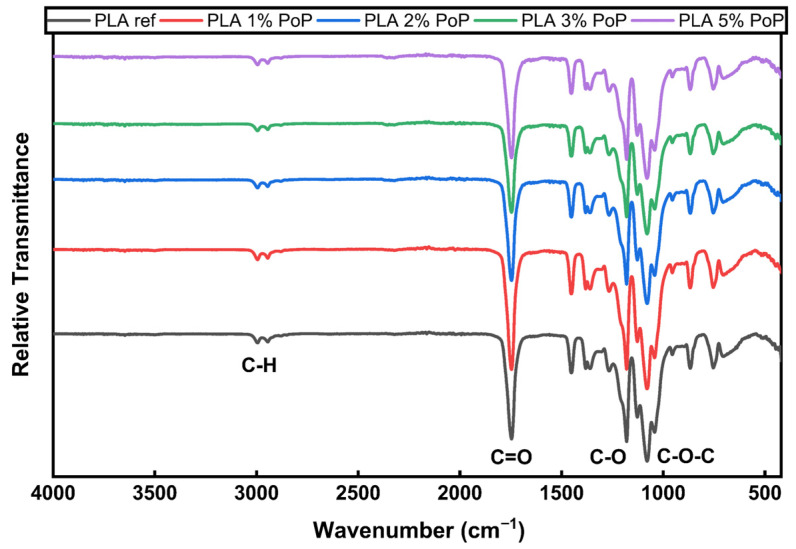
FTIR results of neat PLA and PoP-containing films. Spectra without background and background measurement can be found in the Appendix A.1.

In Figure 1, FT-IR results of pristine PLA and PoP-containing PLA films were presented. The peaks in the range of 3000–2850 cm^−1^ represent the symmetric and asymmetric stretching of aliphatic C–H bonds, from the PLA and PoP [29,30]. A sharp peak around 1750 cm^−1^ is associated with the C=O functional group, a key structural feature of PLA [29]. Additionally, the region between 1300 and 1000 cm^−1^ shows medium-to-strong peaks corresponding to C–O stretching and C–O–C asymmetric vibrations, which indicate ester linkages in the polymer [29].

In PLA films compounded via the melt extrusion process, PoP was incorporated at relatively low concentrations (1–5 wt.%). As can be seen in Figure 1, FT-IR analyses did not reveal any significant spectral differences. However, a slight decrease in the intensity of the characteristic peaks of PLA, such as peak around 1750 cm^−1^, was observed in the PLA 5% PoP sample. The reduced C=O intensity at 1750 cm^−1^ in PLA 5% PoP likely results from a combination of optical dilution by the filler, hydrogen-bonding interactions between PoP phenolics and PLA ester groups, and possible partial hydrolytic/thermal degradation of PLA during high-temperature extrusion. However, FTRI analysis alone does not yield any conclusion for an actual molar mass decrease.

Despite extensive studies on PLA films, a specific knowledge gap persists regarding the detailed influence of different plant fibers and food waste additives on molecular interactions, as determined by FTIR. Several studies have reported the retention of hydroxyl groups or specific functional groups that influence hydrophilicity and compatibility, thereby affecting composite properties [31]. However, some studies have noted minimal or no significant FTIR shifts with certain additives, indicating that physical rather than chemical interactions predominate [32].

It should also be considered that although PoP is inherently rich in polyphenols, tannins, flavonoids, and hydroxyl functional groups, it is likely to undergo significant structural changes when exposed to elevated temperatures (~195 °C) during the melt extrusion process [12]. As a result, in contrast to unprocessed PoP samples, the characteristic –OH stretching bands were not observed in the FT-IR spectra, most likely due to the thermal degradation or transformation of hydroxyl-containing compounds [33].

In summary, FT-IR confirms the characteristic PLA bands, such as C–H at 3000–2850 cm^−1^, C=O near 1750 cm^−1^, and C–O/C–O–C at 1300–1000 cm^−1^, and adding 1–5 wt.% PoP produces no major spectral changes. A comparison to the literature suggests that, in such systems, physical interactions often dominate over new chemical bonding. The absence of –OH stretching bands from PoP likely reflects thermal degradation of hydroxyl-bearing compounds during melt extrusion at 195 °C.

### 3.2. DSC Results

**Figure 2:**
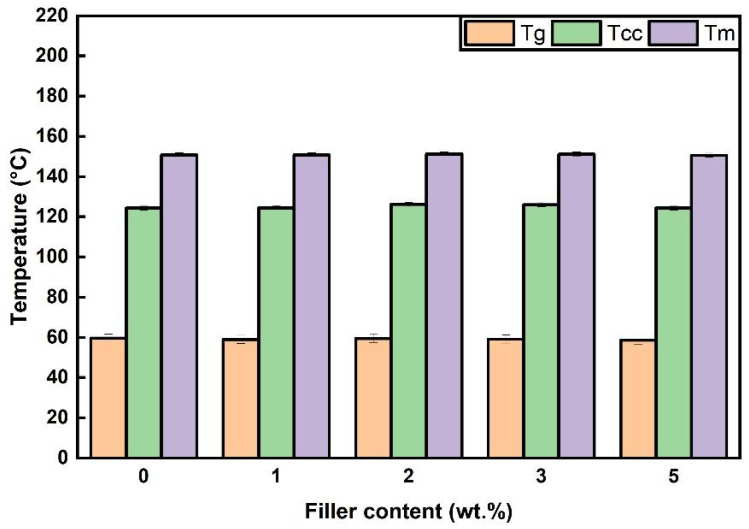
Tg, Tcc, and Tm values of neat PLA and PoP-containing films. DSC curves where values were obtained from are shown in the Appendix A.4.

The DSC analysis of the PLA pellets indicates that the PoP addition into films causes small changes in PLA’s main transition temperatures rather than radically altering them. The neat polymer (PLA ref) exhibits a glass transition temperature (Tg) near 59 ± 2 °C, a cold crystallization temperature (Tcc) around 125 ± 2 °C, and a melting peak temperature (Tm) close to 151 ± 2 °C, as shown in Figure 2. These values are fully consistent with the data sheet of PLA LX175 provided by the producer.

When 1–5 wt.% PoP is incorporated, Tg, Tcc, and Tm values deviated less than 3 °C for each, and the minor fluctuations observed in these characteristic thermal properties are negligible and do not indicate any negative effect. This suggests that the addition of PoP maintains the thermal characteristic of PLA without compromising.

Over the past decade, research has evolved from exploring simple PLA composites to incorporating diverse natural fibers such as flax, jute, rice husk, and food waste residues, aiming to enhance mechanical and thermal properties while reducing environmental impact [34,35,36]. Conversely, the concentration of additives is a critical parameter influencing the thermal behavior of polymeric systems. It has been stated in the literature that bio-additives added to PLA at a rate of over 5 wt.% change the thermal properties of PLA through the chain mobility and crystallization dynamics [37,38]. In this study, the incorporation of PoP at relatively low levels (1–5 wt.%) led to no appreciable changes in the Tg, Tm, or Tcc, suggesting a minimal impact on the segmental mobility and overall thermal transitions of the polymer chains.

In summary, DSC shows that PLA’s main transitions remain essentially unchanged with 1–5 wt.% PoP. The neat PLA has Tg ≈ 59 ± 2 °C, Tcc ≈ 125 ± 2 °C, and Tm ≈ 151 ± 2 °C, which matches the LX175 data sheet. With PoP, Tg, Tcc, and Tm each vary by <3 °C, indicating no meaningful impact on segmental mobility or thermal transitions. This agrees with the literature, which notes that larger shifts typically appear only at concentrations greater than 5 wt.% bio-additive loadings.

### 3.3. Tensile Test Results

As shown in Table 1, PLA ref exhibited a tensile strength of 45.32 ± 5.26 MPa and 1 wt.% PoP remained statistically similar at 47.11 ± 3.49 MPa, which is the same significance group as ref; *p* > 0.05. At higher loadings, strength decreased progressively to 39.20 ± 2.36 MPa, 34.85 ± 2.46 MPa, and 18.37 ± 1.94 MPa, respectively, with increasing PoP concentrations. This trend is consistent with findings reported in the literature; for example, Lyu et al. observed similar reductions in tensile strength when grapefruit seed extract was incorporated into PCL films [39].

A comparable pattern was observed for ductility. PLA ref showed the highest strain at break, which is 0.030 ± 0.003, indicating the highest ductility among the samples. Values decreased upon PoP addition to 0.020 ± 0.004, 0.020 ± 0.001, 0.020 ± 0.002, and 0.010 ± 0.001, respectively.

Young’s modulus showed a modest increase at low loading, followed by a decline at higher PoP levels. The PLA ref showed a modulus of 3037 ± 214 MPa; adding 1 wt.% PoP increased it to 3231 ± 366 MPa, and 2 wt.% PoP yielded 3130 ± 355 MPa. At higher contents, the modulus decreased to 2877 ± 172 MPa with 3 wt.% PoP and further to 2385 ± 310 MPa with 5 wt.% PoP. Thus, any initial stiffening at 1–2 wt.% gives way to a marked reduction in modulus at ≥3 wt.%, aligning with the concurrent losses in strength and elongation.

The combined decrease in tensile strength and strain strongly suggests that the composite is embrittled. Embrittlement in polymer composites is typically due to stress concentration points introduced by rigid filler particles, which hinder the mobility of polymer chains and act as defect points within the structure [20]. Previous studies on PLA composites with additives also highlight similar embrittlement behavior due to rigid particle incorporation and structural defects introduced during processing [19,40,41].

Another critical factor impacting mechanical properties is the filler particle distribution within the PLA matrix. Poor dispersion or agglomeration can amplify the effects of stress concentration and increase the deterioration of mechanical properties [35]. The SEM images in Figure 9 prove that as the PoP concentration increases in the PLA matrix, the agglomerations become visible.

In summary, incorporating PoP into PLA led to noticeable embrittlement, characterized by decreased tensile strength and strain at break. These effects can be primarily attributed to structural defects and reduced polymer chain mobility introduced by the rigid filler particles, filler agglomeration, and possible hydrolytic degradation of PLA during processing, rather than changes in crystallinity.

### 3.4. TGA N_2_ Results

**Figure 3:**
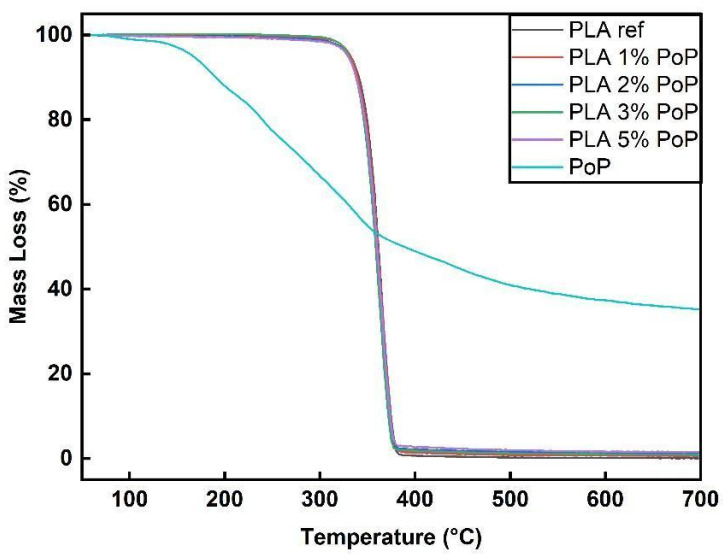
TGA graphs of neat PLA, PoP-containing films, and PoP bio-filler in N_2_ environment.

Focusing on the thermal stability of the bio-filler before discussing the results for the PLA-based films, PoP showed a first mass loss at 100 to 120 °C, as shown in Figure 3 (for DTG curves see Appendix A.3). Natural materials, even after preparation to obtain dry and fine powders, typically contain residual moisture. Accordingly, this mass loss can be attributed to moisture evaporation [42].

However, the first main decomposition step of PoP was around 150 to 180 °C, which is comparable to other fruit by-products, such as grape peels [43]. The multiple degradation steps after 180 °C can be attributed to cellulose, lignin, and hemicellulose in the pomegranate [29,42].

Sucrose thermal degradation occurs by Maillard reaction and caramelization, and the rate of reactions is accelerated by temperature increase, particularly above 100 °C [44]. During the caramelization process, fructose and fructofuranosyl cation may be produced. The fructofuranosyl cation can be transformed into 5-HMF when heated to temperatures exceeding 250 °C [45]. Gurbanov et al. reported that PoP displays a wide and endothermic peak centered around 155 °C, with an additional shoulder at 210 °C [46]. This complex peak encompasses several processes, including water evaporation, melting, dihydroxylation, and decarboxylation, most likely involving pectin, other polysaccharides, and the protein structures found in PoP.

Compared to cellulose and hemicellulose, lignin exhibits a wider range of degradation and, according to Dubey et al., cellulose decomposes from around 310 to 400 °C, hemicellulose breaks down between 210 and 325 °C, and lignin degrades from about 160 °C up to 900 °C [47]. Lignin’s most significant weight loss occurs between 240 and 340 °C [48]. This behavior of lignin is linked with various functional groups in it, such as carbonyl, hydroxyl, carboxyl, phenolic, and methoxy groups [48]. Figure 3 shows that PoP leaves a residue even after 700 °C, as it does not fully decompose. This suggests pomegranates have a high carbon content. The study by Qureshi et al. also supports this argument [49]. 

**Figure 4:**
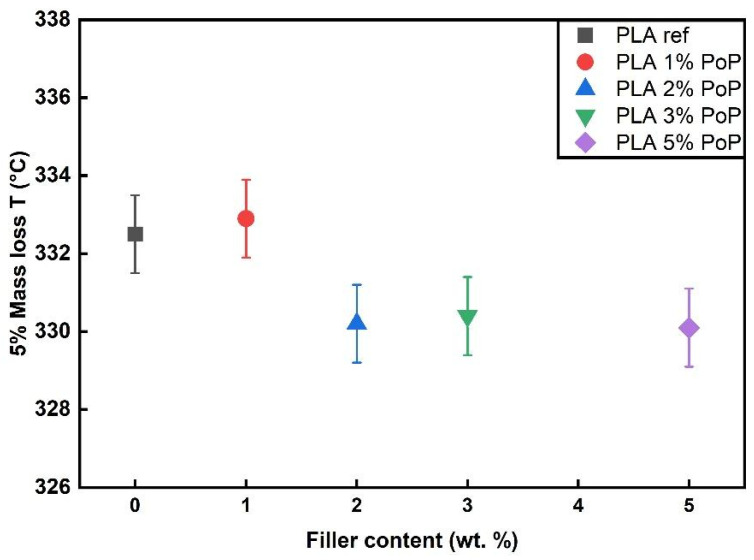
5% mass loss temperature of films in N_2_ environment.

As shown in Figure 3, all film samples followed a similar trend in TGA. Thermal degradation started around 325 to 335 °C, with a main degradation step between 365 and 380 °C. The fact that the graphs of the biocomposite films are almost congruent to the PLA ref indicates that the addition of PoP did not drastically alter the thermal stability of PLA films in a nitrogen environment. The results are in line with findings using wine grape pomace, biogenic by-products from winemaking, in PBS [50]. Especially at filler contents below 5 wt.%, the authors found almost unchanged thermal stability of the biocomposites [50]. Comparable conclusions were drawn for other agricultural wastes, such as coffee parchment [51], wheat bran, and onion peels [52]. Consequently, the 5% mass loss temperature values were very close for all samples, ranging from 330 to 333 °C. As shown in Figure 4, PLA 1 wt.% PoP had the highest thermal stability, while the sample with 5 wt.% PoP had the lowest. A previous study using different wine grape pomaces as bio-fillers reported that low filler contents can have a positive impact on the resulting thermal stability due to mild stabilization effects coming from polyphenols contained in the by-product [50]. However, at higher filler contents, the lower intrinsic thermal stability of the bio-filler, as shown in Figure 3, dominated the thermal behavior. Consequently, slightly reduced 5% mass loss temperatures were obtained for the biocomposites filled with 2, 3, and 5 wt.% PoP, as shown in Figure 4.

Similar to our findings, in the literature, the addition of plant powders or fibers either improved or maintained the thermal stability of PLA films. For example, PLA films with orange peel powder demonstrated a degradation temperature of 546.6 °C, indicating enhanced thermal stability [53]. In another study, the addition of Prosopis Juliflora fiber to PLA increased the thermal stability, as evidenced by TGA results [54]. Furthermore, microcrystalline cellulose from pineapple leaf fibers improved the thermal stability of PLA composites, as evidenced by TGA [55].

In summary, the incorporation of PoP into PLA films did not significantly affect thermal stability, as shown by consistent and stable thermal degradation profiles across all samples, comparable to neat PLA.

### 3.5. TGA O_2_ Results

**Figure 5:**
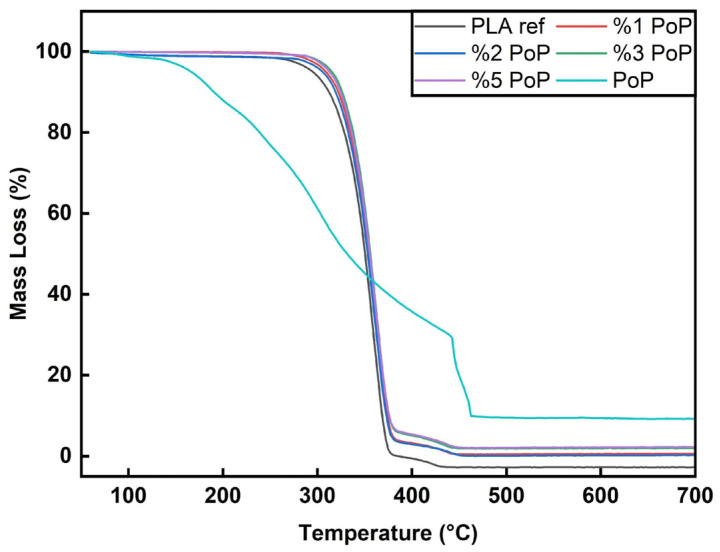
TGA graphs of neat PLA, PoP-containing films, and PoP bio-filler in O_2_ environment.

In addition to TGA measurements in a nitrogen atmosphere, corresponding analyses were conducted in an oxidative environment, as shown in Figure 5 (for DTG curves see Appendix A.3). Starting with the bio-filler, PoP showed a similar degradation behavior in an oxidative as in a nitrogen atmosphere. This is in line with findings from Nanni et al., who investigated the thermal stability of different wine by-products via TGA in different atmospheres [43]. The degradation temperatures in both atmospheres for each by-product were comparable, indicating thermo-oxidative stability of the grape seeds, peels, and stems [43].

**Figure 6:**
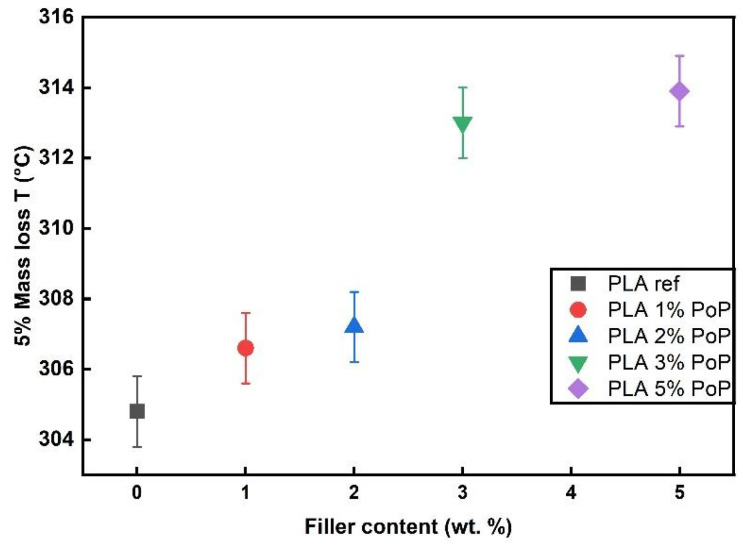
5% mass loss temperature of films in O_2_ environment.

Focusing on the PLA-based samples, PLA ref began to degrade around 280 °C, with a main degradation step between 300 and 400 °C. As shown in Figure 5, PoP-added PLAs started to degrade at slightly higher temperatures around 300 °C. In addition, the main degradation step was also slightly shifted towards higher temperatures, indicating thermo-oxidative stabilization effects coming from the bio-filler. As a result, higher 5 wt.% mass loss temperatures were obtained for the PoP-added samples, as reported in Figure 6.

Moreover, the stability of the samples increased with increasing PoP content, with the highest degradation temperature observed for 5 wt.% PoP. The results are in good agreement with previous studies using wine by-products as biogenic fillers [43,56]. Nanni et al. observed slightly reduced 5% mass loss temperatures for PP filled with 6 wt.% of grape seeds, peels, and skins in a nitrogen atmosphere, whereas about 20 K higher temperatures were obtained for the filled samples in the oxidative atmosphere compared to the neat PP sample [43]. Similarly, wine grape pomace increased the thermo-oxidative stability of PBS with increasing filler content [56]. By adding wine grape pomace in a range of 5 to 20 wt.%, the thermo-oxidative degradation of PBS was suppressed, resulting in higher degradation temperatures obtained from oxidative TGA measurements of the filled samples compared to neat PBS [56]. Accordingly, oxidative TGA in the present study indicates the potential of PoP to act as a thermo-oxidative stabilizer in PLA.

In summary, oxidative TGA shows that PoP behaves similarly in O_2_ and N_2_, and its addition shifts PLA’s degradation to slightly higher temperatures, yielding higher 5% mass loss temperatures, especially at 5 wt.% PoP. These trends match the literature on wine by-product fillers, supporting that PoP can act as a thermo-oxidative stabilizer in PLA.

### 3.6. OIT Results

**Figure 7:**
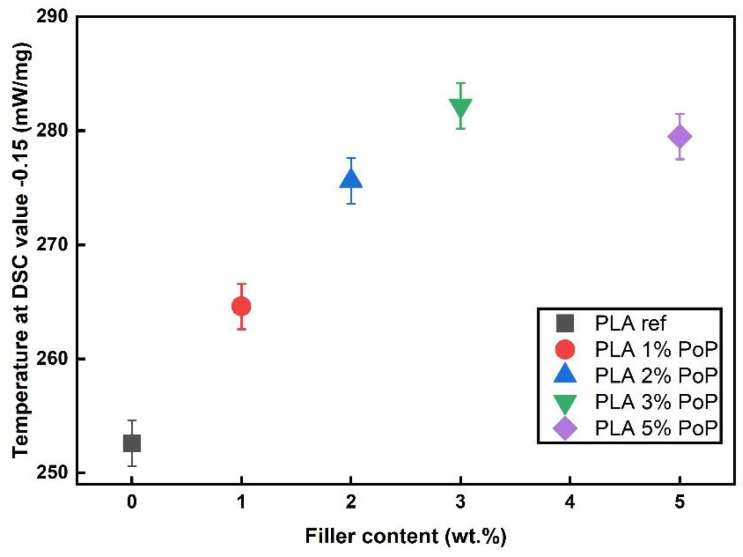
OIT result of neat PLA and PoP-containing films. DSC curves form for OIT measurements are shown in the Appendix A.5.

Dynamic OIT measurements were conducted to verify the thermo-oxidative stabilizing nature of PoP. The accuracy for the determination is ± 2 °C for single measurements. As shown in Figure 7, the addition of PoP led to an increase in OIT of PLA. The stabilization by PoP can be attributed to the natural antioxidants, such as polyphenols, contained in the by-product [50]. In our previous study, the TPC of PoP ethanolic extracts varied from 901.2 to 1042.7 mg GAE/g, DPPH values were 349.1 to 396.1 mM TE/g, and ABTS were 45 to 51.4% [23]. The polyphenols in PoP can scavenge radicals and thereby prevent thermo-oxidative degradation of the PLA, which is based on radical chain reactions [50,51,56]. Maximum thermo-oxidative stability was obtained for the sample filled with 3 wt.% PoP, increasing the degradation temperature by about 30 K. At a filler content of 5 wt.%, a slightly reduced OIT was obtained. A concentration-dependent stabilization effect is well-known for antioxidants in polymers, as an over-concentration leads to pro-oxidant effects and thereby reduces degradation temperatures after an initial increase in stability based on antioxidant effects [57,58]. Accordingly, the OIT measurements verified the oxidative TGA results regarding the potential of PoP for use as a natural antioxidant in PLA. In addition, 3 wt.% appeared as the optimum filler content for thermo-oxidative stabilization of PLA by PoP.

In summary, dynamic OIT tests show that PoP increases PLA’s thermo-oxidative stability via its polyphenol antioxidants. The effect peaks at 3 wt.% PoP and then slightly declines at 5 wt.%, which is consistent with the typical concentration-dependent behavior of antioxidants. These results align with the oxidative TGA findings and identify 3 wt.% as the optimal loading for stabilization.

### 3.7. Color Results

The color values of PLA ref and the PLA films enriched with PoP are reported in Table 2. The L* value decreased with increasing PoP concentration (*p* < 0.05). The PLA ref had the highest lightness (98.19), which means it is the most reflective or closest to white. With 5 wt.% PoP, the L* value decreased to 88.55, indicating a darker color. The a* values of PLA films increased from 0.06 to 2.96 with increasing PoP concentration. The redness of PLA 3 wt.% PoP and 5 wt.% PoP films were found to be similar (*p* > 0.05) and higher than the others. Moreover, there was no significant difference for the a* values of PLA 1 wt.% and 2 wt.% PoP films (*p* > 0.05). The b* values of PLA films enriched with PoP showed an increasing trend, indicating a shift towards yellow. The highest b* value was observed in the PLA 5 wt.% PoP films, and no significant differences were found between 3 wt.% and 5 wt.% PoP addition (*p* > 0.05).

Consistently, the overall color difference (ΔE) rose from 6.1 at 1 wt.% to 16.3 at 5 wt.% PoP, with 1–2 wt.% belonging to the same statistical group and 3–5 wt.% forming a higher, distinct group; thus, even 1 wt.% already yields a clearly perceptible change, while ≥3 wt.% produces a strong, readily noticeable shift in appearance. The addition of PoP to PLA films results in a noticeable transformation in color, making the films darker, more saturated, and taking on a reddish-yellow tone. These color changes are driven by the natural pigments in PoP, such as anthocyanins, tannins, and other phenolic compounds, which act as a natural dye [59,60]. The intensity of the color transformation directly correlates with the concentration of PoP, with higher concentrations imparting more pronounced changes. In addition to its functional role as an additive for biodegradable films, this effect highlights PoP’s potential as a sustainable, natural coloring agent, offering an eco-friendly alternative to synthetic dyes for aesthetic and functional applications in packaging [59,60,61].

In the literature, it was reported that the addition of plant powders affected the optical properties of PLA films. For example, Pueraria lobata root powder reduced the transparency of PLA films, which could be beneficial for UV protection [47]. Moreover, the lightness of citrus peel-based PLA films was also affected, with variations depending on the type of citrus peel used [42].

### 3.8. Rheology Results

**Figure 8:**
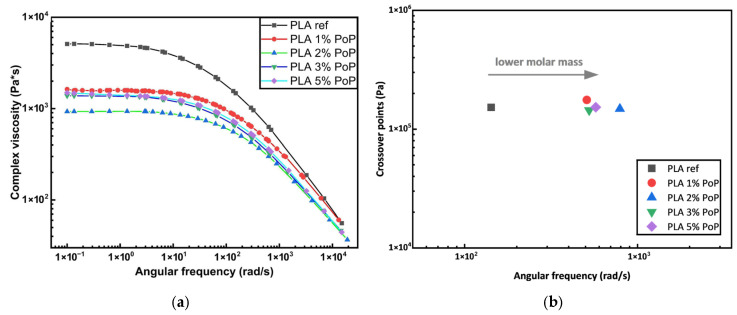
Complex viscosity (**a**) and crossover points (**b**) of PLA films with different wt.% of PoP. Storage and loss modulus as well as tan(δ) against the angular frequency are shown in the Appendix A.1.

The changes in complex viscosity are shown in Figure 8a. Adding PoP to PLA leads to a general decrease in viscosity. For 1 and 2 wt.% of PoP, the complex viscosity drops significantly before increasing for 3 and 5 wt.% of PoP, which is slightly below the level of the viscosity of 1 wt.% PoP. Adding fillers, especially inorganic fillers like silica, usually increases the complex viscosity, particularly in low-frequency regions, and a linear relation between filler concentration and an increase in viscosity can usually be seen. The increase in viscosity when fillers are added originates from an increase in both storage and loss modulus. The storage modulus increases due to limiting chain mobility through more entangled or structured networks. The loss modulus increases due to better energy dissipation during deformation [60]. When looking at fillers from agricultural waste and residues, often the same trend can be seen [61,62]. But this is not the case for the data shown in this work, and there are possibly two reasons why the data differs.

PoP contains, compared to inorganic fillers, higher amounts of fats and oils. These could possibly act as plasticizers. This, however, should also lead to more ductile behavior in tensile testing due to the plasticization and a decrease in T_g_, T_cc_, and T_m_, as well as in degradation temperatures due to an increase in free volume of the polymer chains. As shown above, this is not the case here. Therefore, plasticization cannot adequately explain the loss of viscosity alone.

A decrease in the entanglement of polymer chains also leads to a decrease in viscosity, and entanglement correlates with the chain length. When chains decrease in length through, for example, degradation, entanglement decreases, and hence, complex viscosity decreases also [63]. PoP contains hydrophilic pectin and polysaccharides, and PLA is very susceptible to hydraulic degradation, especially at high temperatures, e.g., during processing or melt rheology measurements [64,65,66]. Bio-additives, including PoP, contain free and bound water. The amount of free water, the moisture content, of PoP was determined to be around 3% during processing. Next to free water, bound water can possibly be released by degradation of bio-additives during processing [67]. The intrinsic thermal stability of PoP is not as high as that of PLA (see Figure 3 and Figure 5). The degradation of PoP starts around 120 °C in oxygen and 110 °C in an inert atmosphere, meaning that additional water is created through the degradation of the filler itself during processing [68]. The proneness of PLA for hydrolytic degradation, as well as the moisture content of around 3% in combination with additional water release through the degradation of PoP during processing, leads to a decrease in molar mass. Figure 8b shows the crossover points of the storage modulus and loss modulus. The crossover points move to higher frequencies without any greater changes in regard to their location on the *x*-axis. This behavior is usually attributed to a decrease in molecular weight or molar mass. This means that hydrolytic degradation during processing, and possibly during rheology measurement, is the main reason for the reduction in viscosity of blends with PoP compared to the reference.

This, however, does not explain the increase in the viscosity for 3 and 5 wt.% PoP up to the viscosity valuesof the blend with 1 wt.% PoP. If the viscosity were solely influenced by hydrolytic degradation, it would further decrease with increasing amounts of PoP. The small increase in viscosity seen in Figure 8 might be attributed to the effect of the filler on the viscoelastic response, where an increase in filler content leads to an increase in viscosity [60,61,62].

In summary, the reduction in viscosity observed upon incorporating PoP into PLA can be attributed to a decrease in molar mass caused by hydrolytic degradation during processing, as was already indicated by FTIR measurements. This degradation is induced by the water present in the PoP, including both free and bound water. The slight increase in viscosity observed at higher PoP contents is likely due to the typical rheological effect of solid fillers, which tend to increase the complex viscosity through particle–matrix interactions.

### 3.9. SEM Results

**Figure 9:**
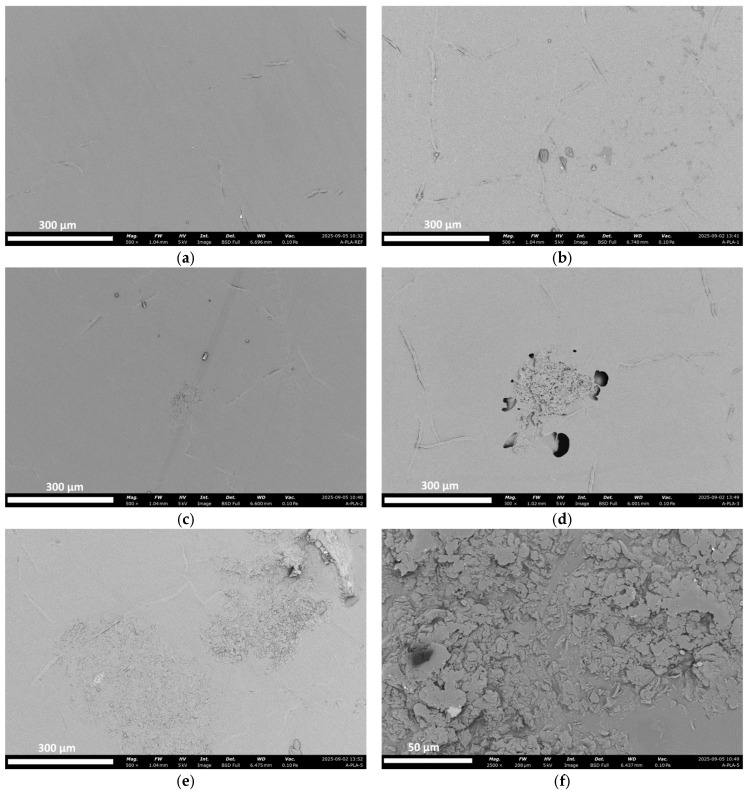
SEM images at 500× magnification showing the surface morphologies of (**a**) PLA ref, (**b**) PLA 1% PoP, (**c**) PLA 2% PoP, (**d**) PLA 3% PoP, (**e**) PLA 5% PoP, and (**f**) PLA 5% PoP (×2500).

At 500× magnification, the PLA ref in Figure 9a shows a smooth and uniform surface without visible defects or porosity, which is consistent with its stable mechanical baseline. With 1 wt.% PoP, the surface remains largely homogeneous, and no agglomerates are evident in the SEM image in Figure 9b. This morphology indicates acceptable dispersion and a mostly intact interface at this loading level, which aligns with tensile data that remains close to the reference range. At 2 wt.%, the first clear clusters appear together with small gaps around particles, creating local spots where stress can build up. Correspondingly, the tensile strength begins to decline. At 3 wt.%, a large agglomerate surrounded by crescent-shaped separation gaps becomes visible, showing filler–matrix separation. The associated tensile results show further reduction in strength. Finally, at 5 wt.%, the surface is covered by several large clusters, which leads to cracks starting early, as evident in Figure 9e. Focusing on these clusters at higher magnifications (×2500) reveals that in the 5% wt. sample in Figure 9f, the PoP particles form agglomerated regions. The presence of these regions is thought to promote crack growth and is related to the sudden drop in stress and strain at rupture.

### 3.10. UV-Vis Spectroscopy Results

**Figure 10:**
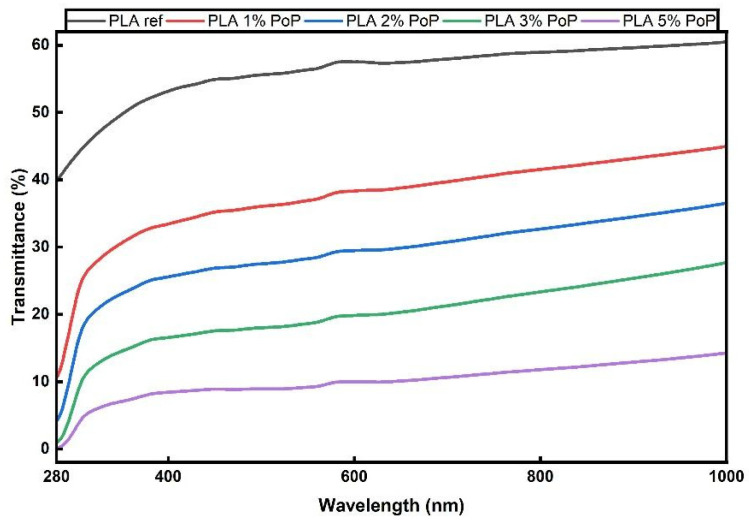
UV-Vis spectroscopy transmittance percentages of PLA films.

The incorporation of PoP produced a clear, concentration-dependent decrease in optical transmittance throughout the spectrum. As evident from Figure 10, at the mid-visible region (≈600 nm), transmittance decreased from ~57–58% for the PLA ref to ~38–40% for 1 wt.% PoP, ~28–30% with 2 wt.% PoP, ~18–20% with 3 wt.% PoP, and ~8–9% for 5 wt.% PoP. Below ~350 nm, 3% and 5% PoP-containing films transmitted only traces of light, evidencing a strong UV-blocking effect attributable to the phenolic content of the filler. This overall behavior aligns with earlier reports on plant-polyphenol stabilizers in PLA, which show that increasing the additive level results in a compromise in visible transparency to achieve UV suppression [18,67]. Colorimetric measurements are fully consistent with spectrophotometry. Relative to the reference film, the lightness coordinate L* decreased from 98.19 ± 0.13 to 88.55 ± 0.49 as PoP increased from 0 to 5 wt.%, confirming progressive darkening of the films (Table 2).

Consequently, when 3–5% PoP was added, UV transmittance was effectively reduced, but the strong increase in chroma and decrease in lightness resulted in a semi-transparent, reddish-yellow-toned film. Color and transmittance values gradually change depending on the percentage of additive. When considered for food packaging, lower additive rates can be used for products requiring shelf visibility, while higher additive rates can be used for products where UV protection is prioritized.

## 4. Conclusions

This study presents the first comprehensive investigation of melt-extruded, solvent-free PLA films incorporating industrial pomegranate peel powder (PoP) as a bio-additive. In contrast to earlier work relying on pomegranate peel extracts or blends produced by solvent casting or electrospinning, this research demonstrates that PoP can be directly integrated into neat PLA under realistic processing conditions (≈195 °C) relevant to industrial film manufacturing.

The systematic characterization across thermal (DSC, TGA in N_2_ and O_2_, and OIT), mechanical, rheological, optical, and morphological domains revealed several key insights. Incorporating PoP with 1–5 wt% did not significantly alter PLA’s characteristic thermal transitions (Tg, Tcc, and Tm), but had a clear effect on its oxidation behavior: thermo-oxidative stability improved markedly, with the OIT increasing by up to 30 K at 3 wt% PoP, confirming the antioxidant potential of phenolic constituents retained after melt processing. At the same time, higher PoP contents (≥3 wt%) led to reduced tensile strength and elongation at break, primarily due to particle agglomeration and hydrolytic chain scission during extrusion. Optical characterization showed a progressive darkening and strong UV-blocking effect, highlighting PoP’s dual role as a natural colorant and UV stabilizer.

Overall, this work closes a critical gap in the literature by linking industrial-scale processing, filler dispersion, and thermo-oxidative stabilization mechanisms for natural, phenolic-rich bio-additives in PLA. The findings establish pomegranate peel powder as a scalable, circular-economy additive that enhances PLA’s functionality without the need for solvent processing or chemical extraction. Future research should focus on improving moisture management to further exploit PoP’s antioxidant potential while mitigating embrittlement.

## Figures and Tables

**Table 1:** Tensile strength, strain at break, and Young’s modulus values of neat PLA and PoP-containing films. Indices a to d indicate significant differences in samples. Stress-strain curves are shown in the Appendix A.6.

Sample	Tensile Strength (MPa)	Strain at Break (ε)	Young’s Modulus (MPa)
PLA ref	45.32 ± 5.26 ^a^	0.03 ± 0.003 ^a^	3037 ± 214 ^a^
PLA 1% PoP	47.11 ± 3.49 ^a^	0.02 ± 0.004 ^b^	3231 ± 366 ^a^
PLA 2% PoP	39.20 ± 2.36 ^b^	0.02 ± 0.001 ^c^	3130 ± 355 ^a^
PLA 3% PoP	34.85 ± 2.46 ^c^	0.02 ± 0.002 ^c^	2877 ± 172 ^b^
PLA 5% PoP	18.37 ± 1.94 ^d^	0.01 ± 0.001 ^d^	2385 ± 310 ^c^

**Table 2:** CIELab color data of neat PLA and PoP-containing films.

Sample	L*	a*	b*	C*	Hue°	Color Difference (ΔE)	Color
PLA ref	98.19 ± 0.13 ^a^	0.06 ± 0.01 ^c^	0.36 ± 0.05 ^d^	0.36	81.19	-	
PLA 1% PoP	94.90 ± 0.22 ^b^	1.11 ± 0.06 ^b^	5.38 ± 0.55 ^c^	5.49	78.39	6.1 ^b^	
PLA 2% PoP	93.34 ± 031 ^c^	1.57 ± 0.12 ^b^	7.76 ± 0.46 ^b^	7.91	78.59	9.0 ^b^	
PLA 3% PoP	90.41 ± 0.95 ^d^	2.58 ± 0.36 ^a^	12.33 ± 1.43 ^a^	12.60	78.17	14.35 ^a^	
PLA 5% PoP	88.55 ± 0.49 ^e^	2.96 ± 0.30 ^a^	13.14 ± 1.15 ^a^	13.47	77.31	16.30 ^a^	

^a–e^ Significant differences in the same column at *p* < 0.05.

**Figure A1:**
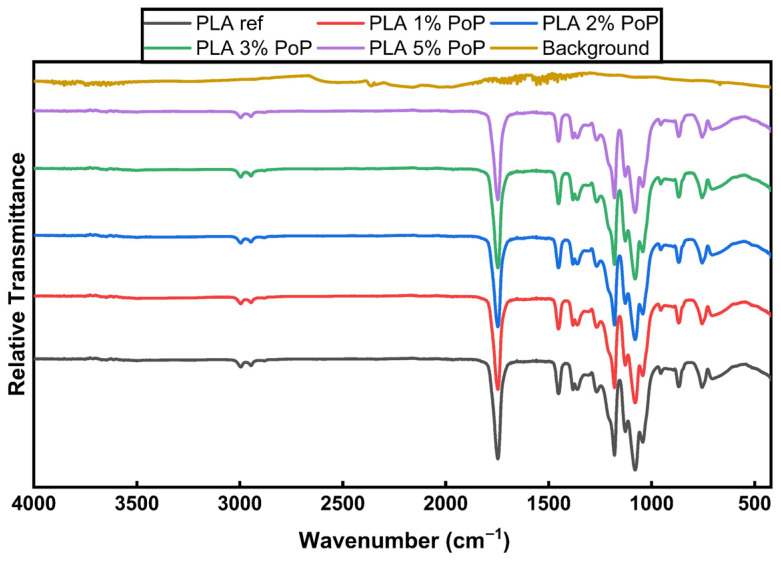
FTIR baseline corrected FTIR spectra, including baseline.

**Figure A2:**
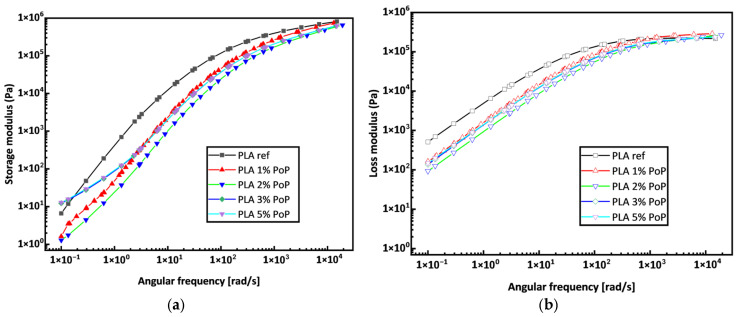
Storage (**a**) and loss modulus (**b**) of PLA films with different wt.% of PoP.

**Figure A3:**
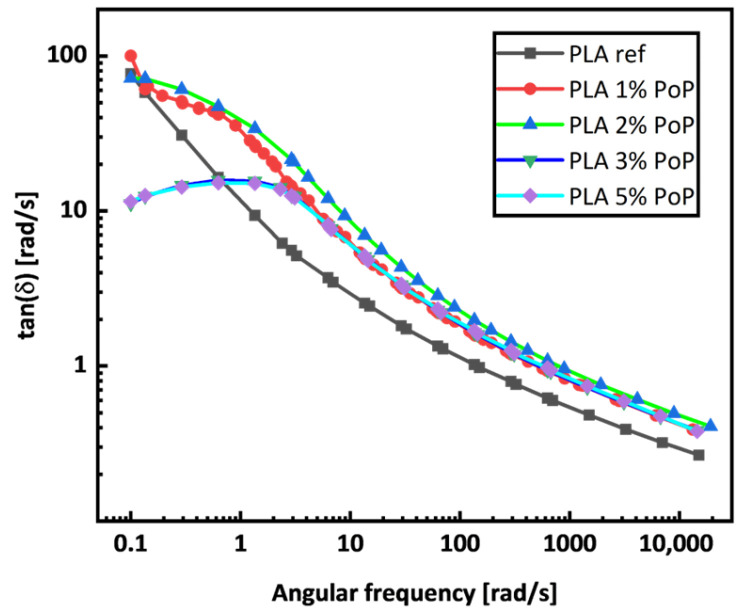
tan(δ) of PLA films with different wt.% of PoP.

**Figure A4:**
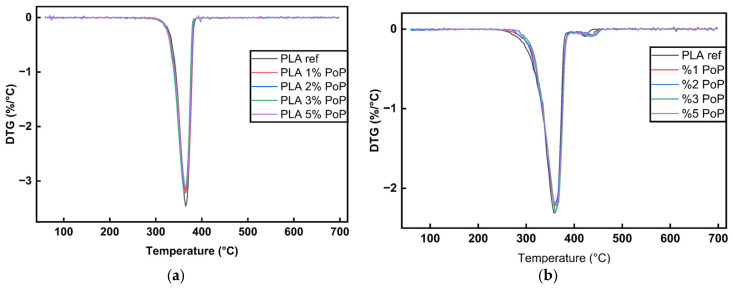
DTG of PLA with different wt.% of PoP in (**a**) nitrogen and (**b**) oxygen atmosphere.

**Figure A5:**
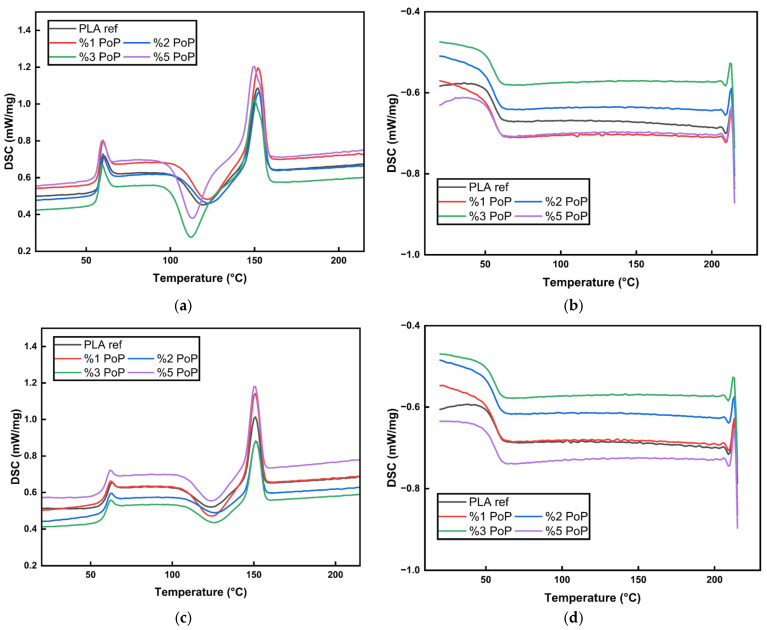
DSC curves of samples: First heating (**a**), first cooling (**b**), second heating (**c**), and second cooling (**d**).

**Figure A6:**
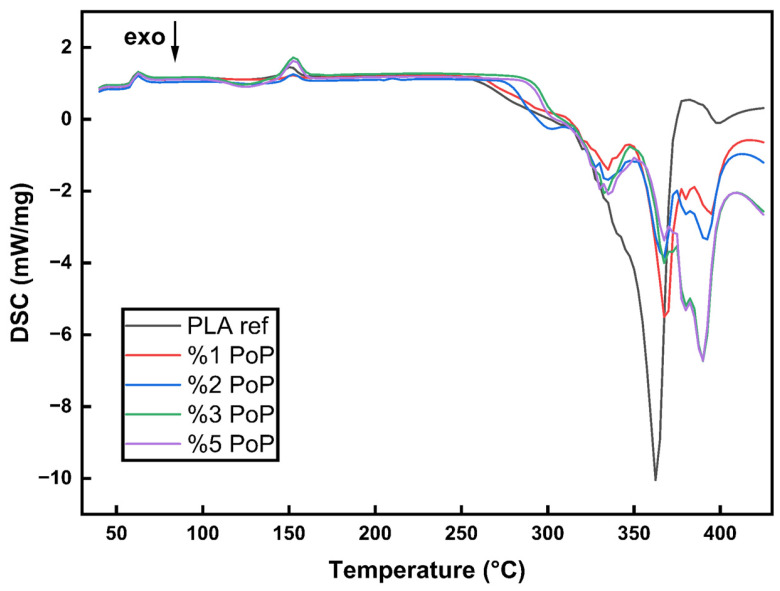
OIT curves of PLA with different wt.% of PoP.

**Figure A7:**
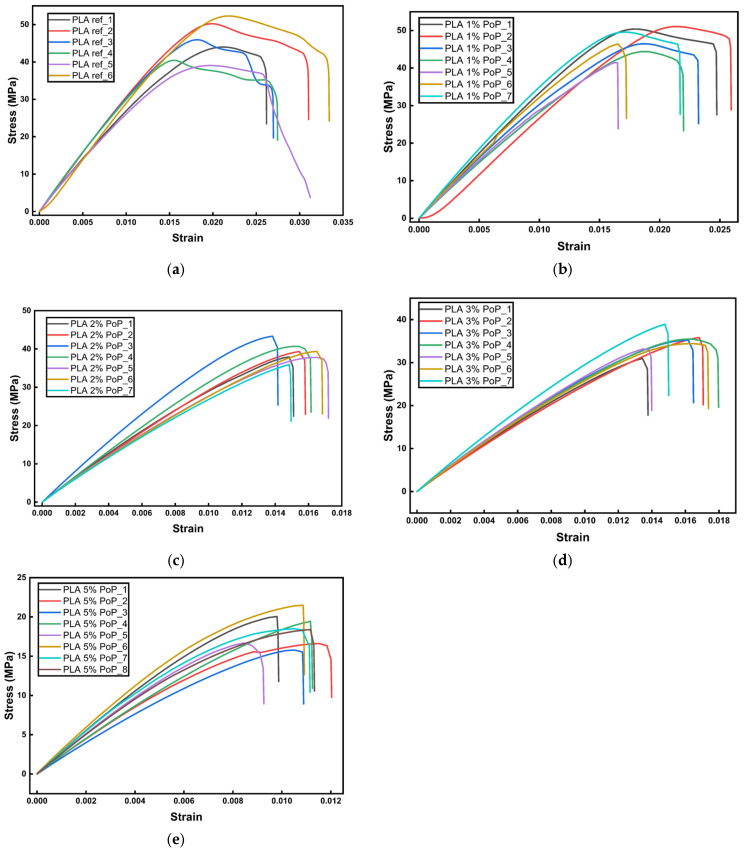
Stress–strain diagrams of PLA with different wt.% of PoP.

## Data Availability

The data presented in this study are available on request.

## Appendix A

### Appendix A.1. FTIR Spectra

**Table A1:** FTIR peak assignment.

Assignment	Peak Position (cm^−1^)
C-H stretch	2950–3000
C=O stretch	1750
C-H stretch	1450
C-O stretch	1100–1200
C-O-C asymmetric stretch	1150–1060

### Appendix A.2. Rheology

The curves for tan(δ) (G″/G′) span from 0.1 to 10,000 rad/s, capturing the full viscoelastic spectrum from viscous-dominated to elastic-dominated behavior (see Figure A3) and exhibit a decrease in increasing angular frequency, which is consistent with the viscoelastic theory and reduced chain relaxation at higher frequencies [69]. For the PLA reference, tan(δ) decreases monotonically, which is consistent with reduced chain relaxation at short time scales. When incorporating PoP, two changes occur: (i) The overall tan δ level decreases, and the position of the tan δ maximum shifts toward higher angular frequencies. This indicates shorter effective relaxation times, which can arise from reduced molecular weight due to hydraulic degradation or restricted chain mobility due to the PoP particles. Both mechanisms are consistent with the observed viscosity reduction at low PoP contents. (ii) There is an increase in tan(δ) for intermediate-to-high frequencies, with the most dramatic increase between the reference and 1 wt.% of PoP. This suggests that interfacial effects dominate, while also indicating that the filler does not act as elastic reinforcement but introduces new mechanisms for energy dissipation instead, or can be attributed to degradation, since shorter chains also further energy dissipation. Agricultural-residue particles typically form broad interphases with imperfect adhesion, generating localized regions where polymer chains undergo additional frictional losses. These dissipative interfaces can outweigh any elastic contribution from the particles, particularly at low filler loadings where a percolating particle network is not expected.

The combined shift in relaxation behavior (tan δ maximum to higher frequencies) and the enhanced high-frequency tan δ, therefore, point toward two concurrent mechanisms: chain scission-induced acceleration of relaxation processes and interfacial damping associated with PoP–PLA interactions.

### Appendix A.3. DTG of TGA

**Table A2:** TGA N_2_ data points.

Sample	Final Residue (%)	T at 5% Mass Loss (°C)	Max T of DTG (Inflection) (1/°C)	T Onset (°C)
PLA ref	0.08	332.5	341.3	347
PLA 1% PoP	0.66	332.9	340.2	345.6
PLA 2% PoP	1.28	330.2	338.6	344.5
PLA 3% PoP	0.85	330.4	337.8	342.1
PLA 5% PoP	1.55	330.1	338.5	343.6

**Table A3:** TGAO_2_ data points.

Sample	Final Residue (%)	T at 5% Mass Loss (°C)	Max T of DTG (Inflection) (1/°C)	T Onset (°C)
PLA ref	0.35	304.8	359.1	328.4
PLA 1% PoP	0.65	306.6	359.3	330.8
PLA 2% PoP	0.28	307.2	360.3	329.5
PLA 3% PoP	2.01	313	359.9	333.9
PLA 5% PoP	2.30	313.9	360.5	331.9

## References

[1] Van de Perre D., Serbruyns L., Coltelli M.-B., Gigante V., Aliotta L., Lazzeri A., Geerinck R., Verstichel S. (2024). Tuning Biodegradation of Poly (lactic acid) (PLA) at Mild Temperature by Blending with Poly (butylene succinate-co-adipate) (PBSA) or Polycaprolactone (PCL). Materials.

[2] OECD Temporary Archive. https://web-archive.oecd.org/temp/2022-08-18/622468-increased-plastic-leakage-and-greenhouse-gas-emissions.htm.

[3] Ge J., Li H., Liu P., Zhang Z., Ouyang Z., Guo X. (2021). Review of the toxic effect of microplastics on terrestrial and aquatic plants. Sci. Total Environ..

[4] Rasal R.M., Janorkar A.V., Hirt D.E. (2010). Poly(lactic acid) modifications. Prog. Polym. Sci..

[5] Milovanovic S., Pajnik J., Lukic I. (2022). Tailoring of advanced poly(lactic acid)-based materials: A review. J. Appl. Polym. Sci..

[6] Aliotta L., Gigante V., Geerinck R., Coltelli M.-B., Lazzeri A. (2023). Micromechanical analysis and fracture mechanics of Poly(lactic acid) (PLA)/Polycaprolactone (PCL) binary blends. Polym. Test..

[7] Narancic T., Verstichel S., Reddy Chaganti S., Morales-Gamez L., Kenny S.T., De Wilde B., Babu Padamati R., O’Connor K.E. (2018). Biodegradable Plastic Blends Create New Possibilities for End-of-Life Management of Plastics but They Are Not a Panacea for Plastic Pollution. Environ. Sci. Technol..

[8] Ashori A., Nourbakhsh A. (2009). Mechanical behavior of agro-residue-reinforced polypropylene composites. J. Appl. Polym. Sci..

[9] Valdés A., Fenollar O., Beltrán A., Balart R., Fortunati E., Kenny J.M., Garrigós M.C. (2016). Characterization and enzymatic degradation study of poly(ε-caprolactone)-based biocomposites from almond agricultural by-products. Polym. Degrad. Stab..

[10] Xie Y., Niu X., Yang J., Fan R., Shi J., Ullah N., Feng X., Chen L. (2020). Active biodegradable films based on the whole potato peel incorporated with bacterial cellulose and curcumin. Int. J. Biol. Macromol..

[11] Suriyaprom S., Mosoni P., Leroy S., Kaewkod T., Desvaux M., Tragoolpua Y. (2022). Antioxidants of Fruit Extracts as Antimicrobial Agents against Pathogenic Bacteria. Antioxidants.

[12] Gullon B., Pintado M.E., Pérez-Álvarez J.A., Viuda-Martos M. (2016). Assessment of polyphenolic profile and antibacterial activity of pomegranate peel (*Punica granatum*) flour obtained from co-product of juice extraction. Food Control.

[13] Karakuş E., Ayhan Z., Haskaraca G. (2023). Development and characterization of sustainable-active-edible-bio based films from orange and pomegranate peel waste for food packaging: Effects of particle size and acid/plasticizer concentrations. Food Packag. Shelf Life.

[14] Shen X., Sun X., Xie Q., Liu H., Zhao Y., Pan Y., Hwang C.-A., Wu V. (2014). Antimicrobial effect of blueberry (*Vaccinium corymbosum* L.) extracts against the growth of Listeria monocytogenes and Salmonella Enteritidis. Food Control.

[15] Wang K., Lim P.N., Tong S.Y., Thian E.S. (2019). Development of grapefruit seed extract-loaded poly(ε-caprolactone)/chitosan films for antimicrobial food packaging. Food Packag. Shelf Life.

[16] Abdel-Naeem H.H.S., Elshebrawy H.A., Imre K., Morar A., Herman V., Pașcalău R., Sallam K.I. (2022). Antioxidant and Antibacterial Effect of Fruit Peel Powders in Chicken Patties. Foods.

[17] Rudra S.G., Gundewadi G., Sharma R.R., Siddiqui M.W. (2020). 8—Natural additives with antimicrobial and flavoring potential for fresh-cut produce. Fresh-Cut Fruits and Vegetables.

[18] Dai L., Li R., Liang Y., Liu Y., Zhang W., Shi S. (2022). Development of Pomegranate Peel Extract and Nano ZnO Co-Reinforced Polylactic Acid Film for Active Food Packaging. Membranes.

[19] Bodbodak S., Shahabi N., Mohammadi M., Ghorbani M., Pezeshki A. (2021). Development of a Novel Antimicrobial Electrospun Nanofiber Based on Polylactic Acid/Hydroxypropyl Methylcellulose Containing Pomegranate Peel Extract for Active Food Packaging. Food Bioprocess Technol..

[20] Li Y., Fu J., Xu Y., Ali A., Hussain Z., Duan Q., Liu H., Yu L. (2024). Antimicrobial packaging materials of PLA/starch composites functionalized by pomegranate peel. J. Taiwan Inst. Chem. Eng..

[21] Muhammad A., Dayisoylu K.S., Pei J., Khan M.R., Salman M., Ahmad R., Ullah H., Noor G.R. (2023). Compositional analysis of natural pomegranate peel powder dried by different methods and nutritional and sensory evaluation of cookies fortified with pomegranate peel powder. Front. Nutr..

[22] Ranjitha J., Bhuvaneshwari G., Terdal D., Kavya K. (2018). Nutritional composition of fresh pomegranate peel powder. Int. J. Chem. Stud..

[23] Çelebi N., Aral N., Taştan Ö. (2024). Development and characterization of pomegranate peel powder and waterborne polyurethane-coated fabrics. J. Coat. Technol. Res..

[24] Ain H.B.U., Tufail T., Bashir S., Ijaz N., Hussain M., Ikram A., Farooq M.A., Saewan S.A. (2023). Nutritional importance and industrial uses of pomegranate peel: A critical review. Food Sci. Nutr..

[25] Mohlamonyane M.J., Adeyemi J.O., Fawole O.A. (2024). Pomegranate fruit peel: A sustainable bioresource in food preservation. Food Biosci..

[26] Hiller B., Rennert M., Nase M. Comparison of the properties of biogenic wine by-products stabilized biocomposites compounded with a miniaturized single-screw extruder and a co-rotating twin-screw extruder. Proceedings of the 60th ISC, Ilmenau Scientific Colloquium.

[27] (2018). Standard Test Method for Tensile Properties of Thin Plastic Sheeting.

[28] Pathare P.B., Opara U.L., Al-Said F.A.-J. (2013). Colour Measurement and Analysis in Fresh and Processed Foods: A Review. Food Bioprocess Technol..

[29] Ben-Ali S., Akermi A., Mabrouk M., Ouederni A. (2018). Optimization of extraction process and chemical characterization of pomegranate peel extract. Chem. Pap..

[30] Siriprom W., Sangwaranatee N., Herman, Chantarasunthon K., Teanchai K., Chamchoi N. (2018). Characterization and analyzation of the poly (L-lactic acid) (PLA) films. Mater. Today Proc..

[31] Massijaya S.Y., Lubis M.A.R., Nissa R.C., Nurhamiyah Y., Kusumaningrum W.B., Marlina R., Ningrum R.S., Sutiawan J., Hidayat I., Kusumah S.S. (2024). Thermal Properties’ Enhancement of PLA-Starch-Based Polymer Composite Using Sucrose. Polymers.

[32] Giani N., Maccaferri E., Benelli T., Bovo M., Torreggiani D., Campari E.G., Tassinari P., Giorgini L., Mazzocchetti L. (2024). Valorization of Agro-Wastes as Fillers in PLA-Based Biocomposites for Increasing Sustainability in Fused Deposition Modeling Additive Manufacturing. Materials.

[33] Tunçalp M., Seki Y., Altay L., Sarikanat M., İsbilir A. (2025). Antimicrobial Performance of PLA/Pomegranate Peel Composites: Effect of Filler Content and Extrusion Parameter, Screw Speed. SPE Polym..

[34] Mu W., Chen X., Li S., Sun Y., Wang Q., Na J. (2023). Mechanical Performances Analysis and Prediction of Short Plant Fiber-Reinforced PLA Composites. Polymers.

[35] Wang L., Yong L.X., Loo S.C.J. (2024). Utilizing Food Waste in 3D-Printed PLA Formulations to Achieve Sustainable and Customizable Controlled Delivery Systems. ACS Omega.

[36] Xing D., Wang H., Tao Y., Zhang J., Li P., Koubaa A. (2025). 3D-printing continuous plant fiber/polylactic acid composites with lightweight and high strength. Polym. Compos..

[37] Miller K., Reichert C.L., Loeffler M., Schmid M. (2024). Effect of Particle Size on the Physical Properties of PLA/Potato Peel Composites. Compounds.

[38] Şen İ., Eroğlu M., Severgün O., Kızıl D. (2024). Ecofriendly quince peel powder incorporated Polylactic acid biocomposite film. J. Elastomers Plast..

[39] Lyu J.S., Lee J.-S., Han J. (2019). Development of a biodegradable polycaprolactone film incorporated with an antimicrobial agent via an extrusion process. Sci. Rep..

[40] Oyeoka H.C., Ewulonu C.M., Nwuzor I.C., Obele C.M., Nwabanne J.T. (2021). Packaging and degradability properties of polyvinyl alcohol/gelatin nanocomposite films filled water hyacinth cellulose nanocrystals. J. Bioresour. Bioprod..

[41] Bo X.-C., Wang D.-C., Liu Y.-X., Li M.-H. (2023). Degradable cellulose/polylactic acid facial masks with antibacterial and antioxidant action from pomegranate extract. Cellulose.

[42] Hashem A., Aniagor C.O., Fikry M., Taha G.M., Badawy S.M. (2023). Characterization and adsorption of raw pomegranate peel powder for lead (II) ions removal. J. Mater. Cycles Waste Manag..

[43] Nanni A., Messori M. (2018). A comparative study of different winemaking by-products derived additives on oxidation stability, mechanical and thermal proprieties of polypropylene. Polym. Degrad. Stab..

[44] Quintas M., Brandão T.R.S., Silva C.L.M. (2006). Modelling autocatalytic behaviour of a food model system- sucrose thermal degradation at high concentrations. J. Food Eng..

[45] Karadeniz F., Atalay D., Erge H.S., Kaya S., Işık B., Aslanali O. (2024). Kinetics of 5-hydroxymethylfurfural (5-HMF) formation and colour change in date fruit fillings stored at different temperatures. J. Food Compos. Anal..

[46] Gurbanov N., Gadimova N., Osmanova S., Ismailov E., Akhundova N. (2022). Chemical composition, thermal stability of pomegranate peel and seed powders and their application in food production. East.-Eur. J. Enterp. Technol..

[47] Dubey S., Kumar R., KumarMondal M. (2024). Pyrolysis kinetics and thermodynamics of pomegranate peel usingTG/DTG analysis. Biomass Convers. Biorefinery.

[48] Li H., Qu Y., Xu J., Fang Z., Smith R.L., Qi X. (2015). Microwave-Assisted Conversion of Lignin. Production of Biofuels and Chemicals with Microwave.

[49] Qureshi W.A., Vivekanandan B., Jayaprasath J.A., Ali D., Alarifi S., Deshmukh K. (2021). Antimicrobial Activity and Characterization of Pomegranate Peel-Based Carbon Dots. J. Nanomater..

[50] Hiller B.T., Schübel L., Rennert M., Krieg D., Nase M., Puch F. (2025). Study of Wine Grape Pomaces from Different Vintages Regarding Their Use as Reliable Sustainable Antioxidants in Biobased Poly(Butylene Succinate). J. Polym. Environ..

[51] Rennert M., Hiller B.T. (2023). Influence of Coffee Variety and Processing on the Properties of Parchments as Functional Bioadditives for Biobased Poly(butylene succinate) Composites. Polymers.

[52] Sasimowski E., Grochowicz M., Szajnecki Ł. (2023). Preparation and Spectroscopic, Thermal, and Mechanical Characterization of Biocomposites of Poly(butylene succinate) and Onion Peels or Durum Wheat Bran. Materials.

[53] As-syirazi A.S., Riantoro G., Syahjaya F.A., Rahmayetty R., Nuryoto N. (2023). Effect of the Addition of Orange Peel Powder on the Physical and Mechanical Properties of Polylactic Acid (PLA)/Cellulose Acetate (CA) Film Composites. J. Rekayasa Kim. Lingkung..

[54] Raj S.S., Kannan T.K., Rajasekar R. (2020). Influence of Prosopis Juliflora wood flour in Poly Lactic Acid—Developing a novel Bio-Wood Plastic Composite. Polímeros.

[55] Moreno G., Ramirez K., Esquivel M., Jimenez G. (2019). Galia Moreno Biocomposite Films of Polylactic Acid Reinforced with Microcrystalline Cellulose from Pineapple Leaf Fibers. J. Renew. Mater..

[56] Hiller B.T., Azzi J.L., Rennert M. (2023). Improvement of the Thermo-Oxidative Stability of Biobased Poly(butylene succinate) (PBS) Using Biogenic Wine By-Products as Sustainable Functional Fillers. Polymers.

[57] Dintcheva N.T., D’Anna F. (2019). Anti-/Pro-Oxidant Behavior of Naturally Occurring Molecules in Polymers and Biopolymers: A Brief Review. ACS Sustain. Chem. Eng..

[58] Dintcheva N.T., Arrigo R., Baiamonte M., Rizzarelli P., Curcuruto G. (2017). Concentration-dependent anti-/pro-oxidant activity of natural phenolic compounds in bio-polyesters. Polym. Degrad. Stab..

[59] Sheng Y.-J., Jiang S., Tsao H.-K. (2007). Effects of geometrical characteristics of surface roughness on droplet wetting. J. Chem. Phys..

[60] Hao X., Kaschta J., Schubert D.W. (2016). Viscous and elastic properties of polylactide melts filled with silica particles: Effect of particle size and concentration. Compos. Part B Eng..

[61] Barczewski M., Mysiukiewicz O. (2018). Rheological and Processing Properties of Poly(lactic acid) Composites Filled with Ground Chestnut Shell. Polym. Korea.

[62] Andrzejewski J., Grad K., Wiśniewski W., Szulc J. (2021). The Use of Agricultural Waste in the Modification of Poly(lactic acid)-Based Composites Intended for 3D Printing Applications. The Use of Toughened Blend Systems to Improve Mechanical Properties. J. Compos. Sci..

[63] Dorgan J.R., Williams J.S., Lewis D.N. (1999). Melt rheology of poly(lactic acid): Entanglement and chain architecture effects. J. Rheol..

[64] Velghe I., Buffel B., Vandeginste V., Thielemans W., Desplentere F. (2023). Review on the Degradation of Poly(lactic acid) during Melt Processing. Polymers.

[65] Jiang N., Li Y., Li Y., Yu T., Li Y., Li D., Xu J., Wang C., Shi Y. (2020). Effect of short jute fibers on the hydrolytic degradation behavior of poly(lactic acid). Polym. Degrad. Stab..

[66] Balli D., Khatib M., Cecchi L., Adessi A., Melgarejo P., Nunes C., Coimbra M.A., Mulinacci N. (2022). Pomegranate peel as a promising source of pectic polysaccharides: A multi-methodological analytical investigation. Food Chem..

[67] Valero L., Gainche M., Esparcieux C., Delor-Jestin F., Askanian H. (2024). Vegetal Polyphenol Extracts as Antioxidants for the Stabilization of PLA: Toward Fully Biobased Polymer Formulation. ACS Omega.

[68] Hiller B.T., Wnuk A., Krieg D., Schübel L., Azzi J.L., Meins T., Nase M., Puch F. (2025). Comparison of the stabilization efficiency of a conventional antioxidant, biobased alternatives derived from wine by-products, and their extracts in poly(butylene succinate) (PBS) and poly(lactic acid) (PLA). Polym. Degrad. Stab..

[69] Dynamic Mechanical Analysis Investigations of PLA-Based Renewable Materials: How Are They Useful?. https://www.mdpi.com/1996-1944/13/22/5302.

